# Data on morphometric analysis of anterior teeth from Hazaribag College of Dental Sciences and Hospital,jharkhand

**DOI:** 10.6026/97320630017060

**Published:** 2021-01-31

**Authors:** Ankur Bhargava, Sonal Saigal, Pragya Thakur, Uddipan Kumar, Shreedevi Bhoi, Shandar Siddiqui

**Affiliations:** 1 Department of Oral Pathology & Microbiology, Hazaribag College of Dental Sciences & Hospital, Hazaribag; 2 Department of Oral Pathology, Microbiology and Forensic Odontology, Dental Institute, Rajendra Institute of Medical Sciences, Ranchi; 3 Department of Conservative Dentistry & Endodontics, Awadh Dental College & Hospital, Jamshedpur; 4 Smile World Dental Hospital, Hazaribag; 5 Department of Oral & Maxillofacial Surgery, Hazaribag College of Dental Sciences & Hospital, Hazaribag;; 6 Department of Pedodontics and Preventive Dentistry, Clinic - Patna Health Care, Patna

**Keywords:** Dimorphism, odontometric, forensic

## Abstract

It is of interest to document data on morphometric (measurement of external form) analysis of maxillary and mandibular anterior teeth collected from a dental set up using mesio-distal (MD) dimension. The mesiodistal dimensions of all permanent anterior teeth (central incisor, lateral incisor and canine) of 25 males and 25 females patients were recorded using digital vernier calipers. Data were charted and statistical analysis was done using Mann Whitney U test. Data shows sexual dimorphism for every tooth between males and females. However, dimorphism was exhibited only in maxillary and mandibular canine, mandibular central incisors, and lateral incisor. Hence,odontometric parameters offer simple, reliable and cost-effective in forensic investigation for recording gender discrimination.

## Background

Seventy percent of the identifications in the event of mass disasters have been confirmed by forensic dentistry [1]. The characteristics of the teeth can remain unchanged even after exposure to extreme environmental conditions, making the tooth an excellent forensic investigative tool [2 - check with authors - Journal discontinued - see PDF version]. Gender dimorphism, refers to those differences in size, stature and appearance between male and female that can be applied to dental identification because no two mouths are alike [3>]. Teeth are readily accessible for examination and since no two teeth have similar morphology, they form an excellent forensic tool for gender determination [4]. The variations in tooth form are a common occurrence and these variations have an ethnic, forensic and anthropological significance [5]. The anterior teeth are esthetically important as they are readily seen during eating, speech, mastication and facial gesticulation. Its size, shape, color and position add to determine and create a definite coherence and order in the arrangement of natural anterior teeth [6]. Gender determination is completed using osteometry, DNA analysis and odontometric parameters. Accurate result is obtained using the DNA analysis. However, it is expensive and not readily available at all locations. It is difficult for DNA requiring qualified trained staff [7]. On the other hand, osteometry is a favoured procedure because it is more effective in determining gender in forensic investigations. However, bodies that are badly mutilated consisting of fragmentary remains of a skeleton are often not trivial for investigation [8]. Odontometric parameters such as mesio-distal and vestibulo-lingual diameters of some permanent teeth show statistically significant differences between men and women [9]. Therefore, It is of interest to document data on morphometric (measurement of external form) analysis of maxillary and mandibular anterior teeth collected from a dental set up using mesio-distal (MD) dimension.

## Methodology

### Dataset nature:

This is a retrospective, cross‑sectional, descriptive study conducted using 50 dental stone models to measure the greatest mesio-distal width of upper and lower anteriors of undergraduate students at a dental college in Hazaribag, India.

### Consent:

Informed consents were obtained from the subjects who included twenty-five male and twenty-five female dental.

### Parameter estimation:

A digital Vernier caliper was used to measure the greatest mesio-distal dimension of the crown of each of the [twelve] teeth investigated, namely the left and right maxillary and mandibular central incisors, lateral incisors and canine. The mesio-distal [MD] dimension has been defined as the greatest distance between the contact points on the proximal surfaces of the dental crown.

### Inclusion criteria:

The study included subjects in age range of 19-23 years with fully erupted teeth, periodontally healthy, non-carious teeth.

### Exclusion criteria:

Subjects with physiological or pathological wearing of teeth (attrition, abrasion, erosion), misaligned teeth (crowding, rotation or malocclusion, spacing), partially erupted teeth, any history of restoration, orthodontic treatment or trauma were excluded from the study sample.

### Data collection:

The width of 25 samples of each type of tooth per gender was measured. In each case, the teeth 11, 12, 13, 21, 22, 23, 31, 32, 33, 41, 42 and 43 were measured (FDI tooth notation). A single examiner to eliminate inter observer error was maintained. All measured dimensions of maxillary and mandibular anteriors are presented as mean and standard deviation (SD). Mann Whitney U test was used for satatistcal analysis.

## Results:

**Figure 1:**
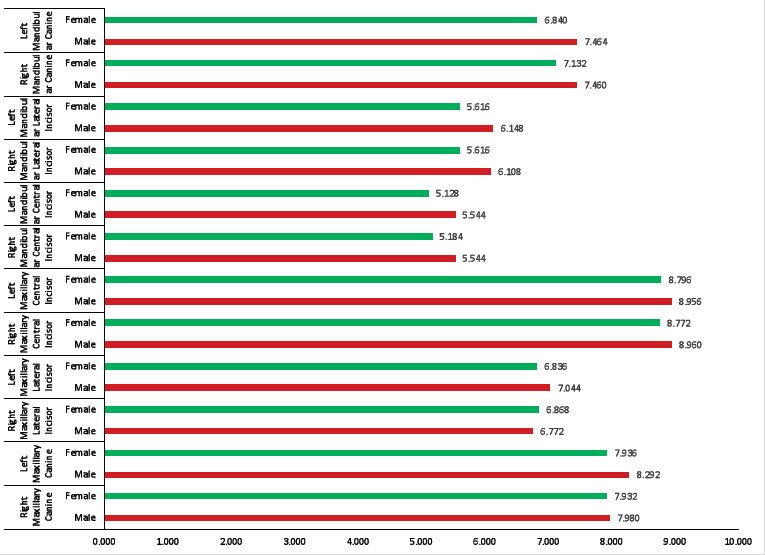
Mean value of mesiodistal dimensions of maxillary and mandibular anterior teeth in male and females.

The morphometric measurements taken from the representative teeth in the maxillary and mandibular series were analyzed statistically for their viability in the expression of values between genders. The mean value of MD dimension of the right maxillary central incisor at the level of contact area was 8.96mm and 8.77mm for male and female, respectively, which was statistically not significant (p>0.05). The left maxillary central incisor was also statistically not significant with the mean value of 8.96 mm and 8.80mm for male and female, respectively (Table 1 - see PDF). But the right and left mandibular central incisor for male and female were highly statistically significant (p<0.001). The mean value of right side was 5.54mm and 5.18mm and left side 5.54mm and 5.13mm for male and female respectively (Table 1 - see PDF,Figure 1). The mean value of the right and left maxillary lateral incisor at the level of contact area was statistically not significant (p>0.05). The mean value of right side was 6.77mm and 6.87mm and left side 7.04mm and 6.84mm for male and female respectively. But result were highly statistically significant (p<0.001) for right and left mandibular lateral incisor with the mean value for right side was 6.11mm and 5.62mm and for left side 6.15mm and 5.62mm male and female respectively (Table 2 - see PDF, Figure 1). The MD dimension for maxillary and mandibular canine result was statistically significant (p<0.01) for both the side. The mean value of MD dimension for right maxillary canine was 7.98mm and 7.93mm and left side 8.29mm and 7.9mm for male and female, respectively. The mean value for mandibular canine in male and female were 7.46 mm and 6.84 mm on the right side and 7.46 mm and 6.84 mm, respectively on the left side (Table 3 - see PDF, Figure 1).

## Discussion:

Gender determination is one of the most important parameter in any forensic investigation. Generally morphological characteristics and anthropometric methods aid in gender determination. Anthropometric method of gender determination usually depends upon available bones and their condition [10] but in case of fragmentary remains use of anthropometric methods is limited. However, odontometric parameters offer an alternative, simple and reliable method for gender determination [11]. Teeth provide excellent material in living and non-living populations for anthropological, genetic, odontological and forensic investigations. The mesiodistal width of the tooth crown is an important axis of morphologic integration [12]. The dimensions are dimorphic among gender, and it has been proved that tooth crowns are larger in men than in women [13]. The mean value of MD dimension of the right maxillary central incisor at the level of contact area was statistically not significant. But for the MD dimension of right and left mandibular central incisor for male and female were highly statistically significant (p<0.001). This is similar to as described elsewhere [14] where the mean width of the right maxillary central incisor for males was found to be 8.944mms and for females 8.613mms. The mean value of the left maxillary central incisor was found out to be 9.056mms for males and 8.664mm for females. Some other studies also show that their statistically significant result in the MD dimension of right and left mandibular central incisor for male and female [15,16]. Very few studies were conducted for MD dimension for lateral incisor for sex determination. In the study of Dash KC [17] right and left maxillary lateral incisor the result were statistically significant with the p value 0.0108 and 0.0009 respectively but for the right and left mandibular lateral incisor result were no significant with the p value 0.2400 and 0.2478. But in our study observation obtained for the maxillary right and left lateral incisor were statistically not significant but for mandibular right and left lateral incisor result statistically significant. This is similar to as described elsewhere [18] where mandibular lateral incisor shows statistically significant in comparison to maxillary lateral incisor in male and female for MD dimension.

**Figure 2:**
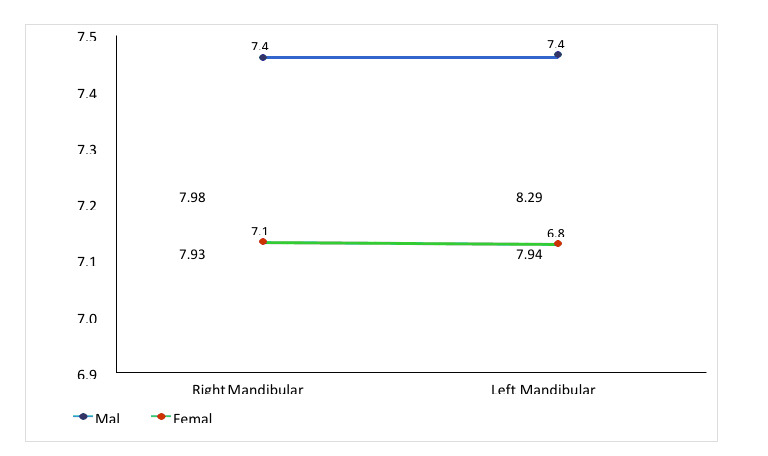
Mean value of mesiodistal dimensions of right and left maxillary canine incisor in male and females.

**Figure 3:**
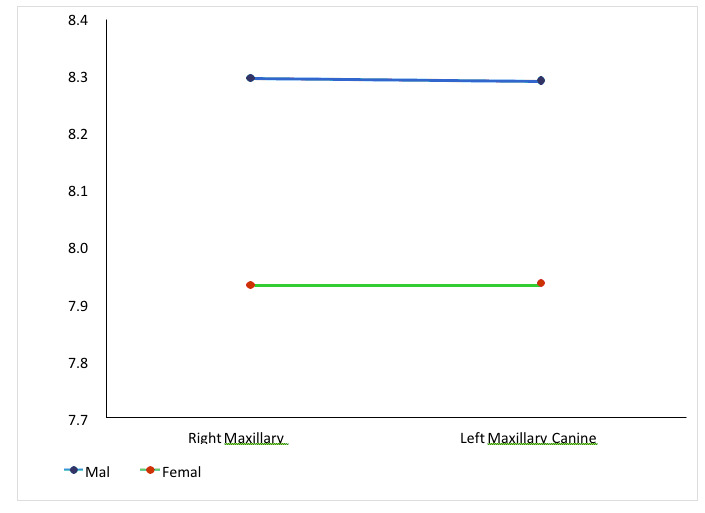
Mean value of mesiodistal dimensions of right and left mandibular canine incisor in male and females.

Recent studies present the canine as the most dimorphic tooth in human dentistry. Mandibular canines are considered reliable elements of human identification, as they are the last teeth to be extracted and are rarely affected by oral diseases and are more likely to survive severe trauma such as an air crash, hurricane or fire [19]. Lebanese subjects a statistically significant difference between men and women p≤ 0.001 in the mesio-distal diameter of the mandibular canine [20]. Similar results of canine dimorphism were also found in other studies. [21,22,23,24]. The MD dimension for maxillary and mandibular canine result was statistically significant (p<0.01) for both the side in male and female. The mean value was higher in male [right side 7.98 and left side 8.29mm] in comparison to female in maxillary teeth (right side 7.93 and left side 7.94mm). Similar results were found for mandibular canine where the mean value was higher in male (right side and left side) in comparison to female canine teeth (right side and left side). The mean value of MD dimension for the both arches of right and left canine of male and female was also not significant (Figure 2-Figure 3). Moreover, very few studies report a significant difference between the right and left side. Saudi population aged 13-20 years showed that the canines were the only teeth to show real dimorphism [25]. They also determined that there was no statistically significant difference between the left and right canines, suggesting that the measurement of teeth on one side could be truly representative when the corresponding measurement on the other side was not possible.

Various theories have been given in the literature for this sexual dimorphism. According to Moss, it is because of the greater thickness of enamel in males due to the long period of amelogenesis as compared to females. However, in females the completion of calcification of the crown occurs earlier in both deciduous and permanent dentition as quoted by de Vito. [26] Gender chromosomes are also known to cause different effects on tooth size. The 'Y' chromosome influences the timing and rate of body development, thus producing slower male maturation, and acts additively and to a greater extent than the 'X' chromosome [26] 'Y' chromosome has a direct effect on tooth size which may be related to a more non-specific effect of hetrero chromatism or cellular activity [27]. The difference in size has been attributed to differently balanced hormonal production between the sexes consequent to the differentiation of either male or female gonads during the sixth or seventh week of embryogenesis rather than any direct effect of gender chromosome themselves [28]. Reason for this dimorphism could be a biologic variation, which is a characteristic of life and is attributed to family, genetics and environmental factors [29]. Variation in food resources exploited by different populations has also been explained as one such environmental cause [27] Previous studies indicate that MD dimensions are more accurate in determining sexual dimorphism [30,31]. These can be useful in archeological, odontologic, genetic, and forensic and crime investigations, as ethnicity/race, culture and environment is known to affect odontometrics. Data shows that gender dimorphism was observed for every tooth included in the study between males and females. Besides this, statistically significant dimorphism was exhibited only in maxillary canine, mandibular anteriors i.e. central incisors, lateral incisor and canines. Linear dimensions of the tooth act as an excellent parameter, which is a simple, affordable, and reliable method for gender determination from the dental remains. Using MD dimensions, the gender dimorphism becomes far better and accurate. Further studies can be done to procure extended data, which can be used by forensic experts as adjuncts to establish gender dimorphism in mass disasters.

## Conclusion

We document data on morphometric analysis of anterior teeth from a dental college in Hazaribag, India to help in forensic investigation.
